# Variability of antibiotic susceptibility and toxin production of *Staphylococcus aureus* strains isolated from skin, soft tissue, and bone related infections

**DOI:** 10.1186/1471-2180-13-188

**Published:** 2013-08-08

**Authors:** Haziz Sina, Théodora A Ahoyo, Wardi Moussaoui, Daniel Keller, Honoré S Bankolé, Yves Barogui, Ymkje Stienstra, Simeon O Kotchoni, Gilles Prévost, Lamine Baba-Moussa

**Affiliations:** 1 Laboratoire de Biologie et de Typage Moléculaire en Microbiologie; Faculté des Sciences et Techniques/Université d’Abomey-Calavi, Cotonou 05 BP 1604, BENIN; 2 EPAC/ Université d’Abomey-Calavi, Cotonou 01 BP 526, BENIN; 3 Unité: EA-4438 Physiopathologie et Médecine, Institut de Bactériologie, 3 rue Koeberlé, Strasbourg F-67000, France; 4 Laboratoire National de Santé Publique, Cotonou, Bénin; 5 Programme National de Lutte contre l'Ulcère de Buruli, Cotonou, Benin; 6 Department of Internal Medicine/Infectious Diseases, University of Groningen, University Medical Center Groningen, P.O. Box 30.001, Groningen 9700 RB, The Netherlands; 7 Department of Biology and Center for Computational and Integrative Biology, Rutgers University, Camden NJ 08102, USA

**Keywords:** *S. aureus*, MRSA, PVL, Pyomyositis, Osteomyelitis, Skin infections, Benin, Africa

## Abstract

**Background:**

*Staphylococcus aureus* is an opportunistic commensal bacterium that mostly colonizes the skin and soft tissues. The pathogenicity of *S. aureus* is due to both its ability to resist antibiotics, and the production of toxins. Here, we characterize a group of genes responsible for toxin production and antibiotic resistance of *S. aureus* strains isolated from skin, soft tissue, and bone related infections.

**Results:**

A total of 136 *S. aureus* strains were collected from five different types of infection: furuncles, pyomyositis, abscesses, Buruli ulcers, and osteomyelitis, from hospital admissions and out-patients in Benin. All strains were resistant to benzyl penicillin, while 25% were resistant to methicillin, and all showed sensitivity to vancomycin. Panton-Valentine leukocidin (PVL) was the most commonly produced virulence factor (70%), followed by staphylococcal enterotoxin B (44%). Exfoliative toxin B was produced by 1.3% of the strains, and was only found in isolates from Buruli ulcers. The *tsst-1*, *sec*, and *seh* genes were rarely detected (≤1%).

**Conclusions:**

This study provides new insight into the prevalence of toxin and antibiotic resistance genes in *S. aureus* strains responsible for skin, soft tissue, and bone infections. Our results showed that PVL was strongly associated with pyomyositis and osteomyelitis, and that there is a high prevalence of PVL-MRSA skin infections in Benin.

## Background

*Staphylococcus aureus* is an opportunistic pathogen that mainly colonizes the nares and skin of up to 80% of the population [1]. *S. aureus* is a Gram-positive cocci that is frequently isolated in hospitals, and is responsible for diverse infections and toxicoses [2]. *S. aureus* is the most common cause of skin and soft-tissue infections (such as impetigo, furunculosis, and abscess), as well as systemic infections (such as pneumonia and endocarditis) [3]. The threat of *S. aureus* is not only due to its distribution and pathogenicity [4,5], but also because of its ability to overcome antimicrobial agents [6-8].

Virulence factors produced by *S. aureus* render this organism highly pathogenic. The known virulence factors include exotoxins, such as exfoliative toxins (ETs), along with toxic shock syndrome toxin-1 (TSST-1), staphylococcal enterotoxins (SEs), leukocidins (Panton-Valentine leukocidin; PVL, LukE/D), and hemolysins (α, β, γ, δ) [9]. Enterotoxins often cause food poisoning [10], while ETs (also called epidermolysins) act on the skin [11].

Among the leukocidins, PVL is an extracellular protein consisting of two subunits, F and S, which act in concert and have leucocidal and dermonecrotic functions. The PVL toxin targets the outer membrane of polymorphonuclear cells, monocytes, and macrophages [12-15]. *S. aureus* strains that are positive for PVL are usually associated with skin and soft-tissue infections, and were first isolated in the 1960s [16-19]. PVL-positive strains are particularly associated with furuncles, accounting for 96% of cases [11,17,20], and approximately 90% of PVL-positive *S. aureus* strains were originally isolated from furuncles. PVL has also been associated with severe infections, including necrotizing pneumonia [19,21-24], osteomyelitis [25], and even cases of purpura fulminans [26]. PVL toxin was recently identified in Lemierre’s syndrome [27], and in a case of Fournier’s gangrene [28]. PVL has also been associated with community-acquired necrotizing and hemorrhagic pulmonary infections affecting previously healthy children and young adults [22,29].

Several antibiotics were routinely used in the treatment of *S. aureus* infections, contributing to the emergence of antibiotic-resistant strains. Widespread resistance severely complicates management of *S. aureus* infections. *S. aureus* strains that are resistant to methicillin (methicillin-resistant *S. aureus*, MRSA) are pervasive in the hospital environment, and have recently also caused a global epidemic of community-associated *S. aureus* (CA-MRSA) infections [30]. The changing trend of MRSA epidemiology, showed the use of PVL locus detection as a marker of CA-MRSA isolates, alongside with non multiresistant pattern and SCCmec type IV or V [31]. Vancomycin has been used successfully for over 50 years for the treatment of *S. aureus* infections, particularly those caused by MRSA strains [32]. However, vancomycin-resistant *S. aureus* (VRSA) and vancomycin-intermediate (VISA) strains have been reported, three decades after the introduction of vancomycin [33]. The presence of resistance genes may also affect toxin production.

The production of multiple virulence factors, as well as the presence of antibiotic resistance genes, makes *S. aureus* a highly pathogenic microorganism. The objective of this work was to study the susceptibility profile and toxin production of *S*. *aureus* strains isolated from various skin, soft tissue, and bone infections.

## Results

### Prevalence of *S. aureus* strains according to the sample origin

Using standard microbiological methods for identification of microorganisms; a total of 136 strains of *S. aureus* were collected during this study. The proportions of the strains varied depending on the five types of infection: furuncle, osteomyelitis, pyomyositis, abscess, and Buruli ulcer. Almost 37% (50/136) of the collected strains originated from abscesses, followed by strains isolated from pyomyositis patients (27%, 37/136), furuncles (14%, 19/136), Buruli ulcers (12%, 16/136), and osteomyelitis cases (10%, 14/136).

### Susceptibility to antibiotics

**Figure 1:**
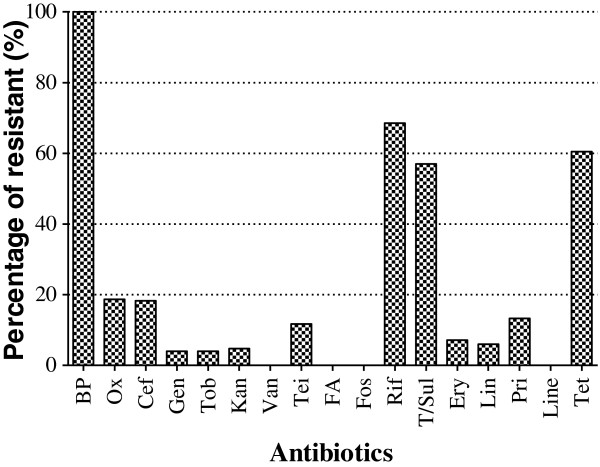
**Global *Staphylococcus aureus *strains isolated from primary and secondary infections resistance profile to 22 antibiotics.** Benzyl penicillin (BP), oxacillin (Ox), cefoxitin screen (Cef), gentamicin (Gen), tobramycin (Tob), kanamycin (Kan), vancomycin (Van), teicoplanin (Tei), fusidic acid (FA), fosfomycin (Fos), rifampicin (Rif), trimethopim/sulfamethoxazole (T/Sul), erythromycin (Ery), lincomycin (Lin), pristinamycin (Pri), linezolid (Line), tetracyclin (Tet).

There was a wide range in the susceptibility of the isolates to the various antibiotics examined. All of the strains were resistant to benzyl penicillin, while other antibiotics (vancomycin, fusidic acid, fosfomycin, and linezolid) were active against some of the strains (Figure 1).

**Figure 2:**
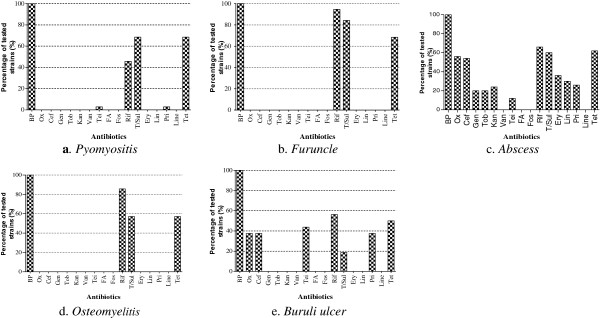
***Staphylococcus aureus *strains resistance profile to 22 antibiotics according to their origin.** Benzyl penicillin (BP), oxacillin (Ox), cefoxitin screen (Cef), gentamicin (Gen), tobramycin (Tob), kanamycin (Kan), vancomycin (Van), teicoplanin (Tei), fusidic acid (FA), fosfomycin (Fos), rifampicin (Rif), trimethopim/sulfamethoxazole (T/Sul), erythromycin (Ery), lincomycin (Lin), pristinamycin (Pri), linezolid (Line), tetracyclin (Tet).

There was no significant difference in the antibiotic resistance of the strains based on their origin (Figure 2). *S. aureus* strains originating from pyomyositis (Figure 2a), furuncles (Figure 2b) and osteomyelitis cases (Figure 2c) were resistant to 4/17 tested antibiotics (benzyl penicillin, rifampincin, tetracycline, and trimetroprim/sulfamethoxazol), while strains originating from abscesses and Buruli ulcer were strongly resistant to respectively 13/17 and 7/17 of the tested antibiotics (Figure 2d, e). Of the 136 isolated *S. aureus* strains, 34 (25%) were resistant to oxacillin (MRSA), while none of the strains showed resistance to vancomycin (VRSA). The oxacillin-resistant strains were all isolated from abscesses and Buruli ulcers.

### Toxins production and/or presence of their encoding genes

**Figure 3:**
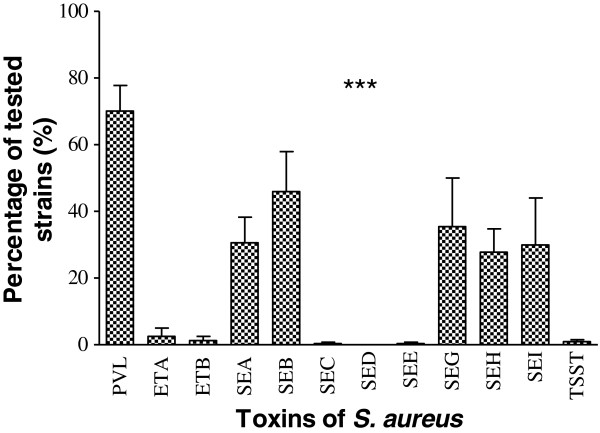
**Toxins production by the *Staphylococcus aureus *strains isolated from primary and secondary infections. PVL**: Panton-Valentine Leukocidin; **ETA**: Exfoliative Toxin A; **ETB**: Exfoliative Toxin B; **SEA**: staphylococcal enterotoxin A; **SEB**: staphylococcal enterotoxin B; **SEC**: staphylococcal enterotoxin C; **SED**: staphylococcal enterotoxin D; **SEE**: staphylococcal enterotoxin E; **SEG**: staphylococcal enterotoxin G; **SEH**: staphylococcal enterotoxin H; **SEI**: staphylococcal enterotoxin I; **TSST**: Toxic-shock syndrome Toxin. Means ± standard deviations (SD) for three experiments are given. ***: p˂0.0001.

**Figure 4:**
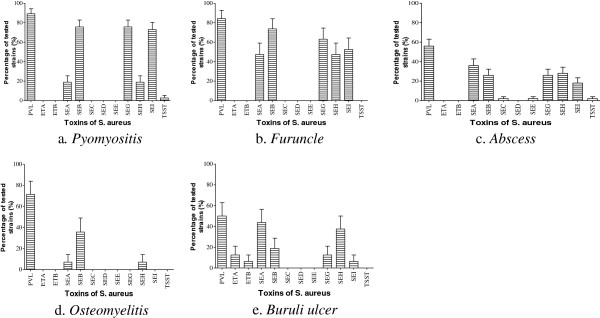
**Specificity of the toxins production by the *S. aureus *strains isolated from primary and secondary infections. PVL**: Panton-Valentine Leukocidin; **ETA**: Exfoliative Toxin A; **ETB**: Exfoliative Toxin B; **SEA**: staphylococcal enterotoxin A; **SEB**: staphylococcal enterotoxin B; **SEC**: staphylococcal enterotoxin C; **SED**: staphylococcal enterotoxin D; **SEE**: staphylococcal enterotoxin E; **SEG**: staphylococcal enterotoxin G; **SEH**: staphylococcal enterotoxin H; **SEI**: staphylococcal enterotoxin I; **TSST**: Toxic-shock syndrome Toxin. Means ± standard deviations (SD) for three experiments are given.

**Figure 5:**
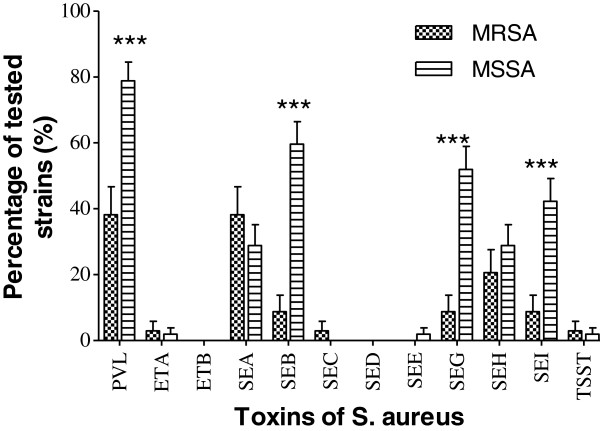
**Effect of Methicillin resistance on the encoding toxins genes presence. PVL**: Panton-Valentine Leukocidin; **ETA**: Exfoliative Toxin A; **ETB**: Exfoliative Toxin B; **SEA**: staphylococcal enterotoxin A; **SEB**: staphylococcal enterotoxin B; **SEC**: staphylococcal enterotoxin C; **SED**: staphylococcal enterotoxin D; **SEE**: staphylococcal enterotoxin E; **SEG**: staphylococcal enterotoxin G; **SEH**: staphylococcal enterotoxin H; **SEI**: staphylococcal enterotoxin I; **TSST**: Toxic-shock syndrome Toxin. Means ± standard deviations (SD) for three experiments are given. ***: P˂0.001; the other differences were not statistically significant (P˃0.05).

There was a significant difference in the production and/or the presence of genes encoding the 12 toxins (p < 0.0001). Thus, a significant number of strains (70.0%) were capable of producing PVL, followed by the production of staphylococcal enterotoxin B (SEB) (44.3%). None of the strains contained the genes responsible for exfoliative toxin B (ETB) or staphylococcal enterotoxin D (SED) production, while the ability to produce staphylococcal enterotoxins C and E (SEC, SEE), as well as the toxic shock syndrome toxin (TSST), was detected in <1% of strains (Figure 3). The observed difference was related to the origin of the *S. aureus* strains. PVL was the most commonly produced toxin, regardless of the origin of the strains (Figure 4). PVL toxin was particularly prevalent in strains isolated from furuncles (89.5%) and pymyositis patients (89.2%). Other toxins were produced in various proportions depending on the origin of the strain (p < 0.0001). There was a significant difference in the detection of genes encoding toxins in MRSA strains (Figure 5).

## Discussion

The *S. aureus* stains analyzed in this study displayed a wide range of sensitivity to the 17 tested antibiotics. Generally, benzyl penicillin was not efficient in controlling the strains (Figure 1). This is consistent with previous reports showing high rates of *S. aureus* resistance (>90%) to benzyl penicillin [34,35], suggesting that this antibiotic, one of the first to be introduced, is no longer effective against *S. aureus*[36]. A very high proportion of strains showed resistance to rifampicin (67%), tetracycline (60%), and trimetroprim/sulfamethoxazol (57%). This finding is consistent with previous studies performed in Africa [37-40]. The high proportion of strains showing resistance to penicillin and three other antibiotics may be explained by the practice of patient self-medication in Benin, and by the availability and low price of the antibiotics. These antibiotics can be bought without prescription, especially in developing countries. In our *in vitro* study, 4/17 tested antibiotics (vancomycin, fusidic acid, fosfomycin, and linezolid) were effective against all the *S. aureus* strains.

The collection of strains isolated from abscesses was sensitive to only four of the 17 tested antibiotics. However, strains isolated from furuncles (Figure 2b) and osteomyelitis patients (Figure 2d) were sensitive to 13 antibiotics (Figure 2). This result can likely be attributed to the origin of the strains. In our study, the samples collected from furuncles and osteomyelitis patients were from an extra-hospital community origin. Indeed, the selection pressure observed when using antibiotics in a hospital environment causes nosocomial strains to develop multi-resistance, in contrast to strains of community origin.

Of the 136 tested strains, 34 (25%) were resistant to oxacillin. This proportion of resistant strains appears to have increased steadily in Benin, compared with the recorded resistance rate of 11.6% in 1999 [40]. However, the overall proportion of oxacillin-resistant strains in Benin is still low compared to surrounding countries [41], even though methicillin is the preferred antibiotic for treatment of staphylococcal infections [42]. To efficiently control MRSA, vancomycin is recommended [43], and in our study we observed no resistance to vancomycin. Fortunately, vancomycin remains active against methicillin resistant strains of *S. aureus*.

In the current study of *S. aureus* strains isolated from skin, soft tissue, and bone related infections, PVL was the most prevalent toxin in our collection (70.0% of strains), followed by SEB (44.3%), SEG (35.5%), SEA (32.0%), SEH (28.8%), and SEI (28.9%). The genes encoding ETB and SED were not detected in any of the strains, while the SEE (0.8%), SEC (0.6%), and TSST (1%) genes were detected, but at a very low rate (Figure 3). This high detection frequency of the gene encoding PVL (p < 0.0001) was observed throughout the analysis, regardless of the origin of the sample (Figure 4). PVL appears to be a primordial toxin of *S. aureus* strains associated with skin, soft tissue, and bone related infections. These results are lower than the 96% of PVL-positive production strains we observed among *S. aureus* isolated from furuncle [20]. But the prevalence of PVL-positive *S. aureus* obtained in our study is higher than the 52.1% observed in Nigeria [44] and in cape Verdes Island [45]. The observe differences observed can be explain by the fact that in our study, we use various kind of strains. However, our result is close to the 72% obtained in Algeria [46]. Then, comparing with the other studies, we can say that the prevalence level of PVL ocus varies with geographical location, and clinical specimen [47] In the clinical field, PVL-positive *S. aureus* strains are more pathogenic than PVL-negative strains [22]. This is explained by the fact that the lytic activity of PVL directly affects monocytes, macrophages, polynuclear neutrophils, and metamyelocytes, although erythrocytes are not lysed by PVL [48]. PVL toxin is known to have a cytolytic effect, and as such polynuclear neutrophils were identified as important indicators of staphylococcal virulence [16]. Moreover, the cytolytic activity of PVL is observed at high toxin concentrations, while apoptosis is observed at low concentrations [49].

Regarding the ETs, only the gene encoding epidermolysin A (ETA) was detected, and in all cases the *S. aureus* strains were isolated from Buruli ulcers (Figure 4e). Such specific production of ETA by Buruli ulcers may be explained by the fact that ETs are known to be serine active proteases, with their activity highly specialized for desmoglein-1, an important epidermal protein [50,51]. Therefore, the production of ETA by *S. aureus* strains from Buruli ulcers indicates a secondary role for *S. aureus* in the development of these ulcers, which are predominantly caused by *Mycobacterium ulcerans*.

Of the eight genes encoding enterotoxins, three (SEC, SED, and SEE) were not detected at all, while three others (SEA, SEB, and SEH) were detected at various proportions, regardless of the origin of the strains (Figure 4). Based on the homology of their sequences, the three detected enterotoxin genes belong to three different groups [52]. This explains the diversity of enterotoxins produced by *S. aureus* isolated from skin infections. Those toxins associated with food poisoning have antigenic and emetic activities [53-55]. The presence of enterotoxin genes in the strains isolated from skin, soft tissue, and bone related infections may be explained by human or environmental contamination, through the presence of open wounds. Similar observations are reported for TSST-1, which is the most prevalent toxin in cases of food poisoning [56].

Our study revealed that resistance to methicillin negatively correlates with toxin production (Figure 5). The difference in toxin production was extremely significant for PVL and some enterotoxins (B, G, and I) (p < 0.0001), and we observed that MSSA strains produced twice as many toxins as MRSA strains. These results suggest that the isolated strains were in majority Hospital acquired methicillin resistance *S. aureus* (HA-MRSA) because the community-acquired methicillin resistance *S. aureus* (CA-MRSA). Indeed, these CA-MRSA have an SCCmec type IV cassette conferring resistance to methicillin [57], and 77% of them harbor genes for Panton- Valentine leukocidin (PVL) [58,59]. In addition, the prevalence of the genes for some toxins is higher in CA-MRSA than in HA-MRSA, suggesting that strains circulating in the community are more toxinogenic than hospital-associated strains [60]. Focusing on the duality of the observed activity between the resistance to methicillin and detection of the PVL-encoding gene, we may deduce that the resistance gene has a repressive activity against PVL. This observation was also made by Baldwin and Lowe [61], and mostly relates to HA-MRSA strains. In addition, we found that the presence of the methicillin resistance gene negatively impacts the expression of the gene encoding PVL. The emergence of MRSA in the hospital acquired strains may be viewed as disadvantageous in the selection of strains producing toxins, notably PVL. Indeed, *mecA*-encoded methicillin resistance involves β-lactamase production [62], which is not favorable for bacterial development [63]. Although community-acquired MRSA infections are increasingly frequent, the use of alternative antibiotics, such as vancomycin or ofloxacin/ciprofloxacin, is not appropriate because of the risk of the development of resistance to these antibiotics. Vancomycin is usually not available because of high costs and the necessity for assessing drug levels in the blood. Studies on the use of vancomycin for prophylaxis in medical centers with high rates of MRSA show that the use of this antibiotic is controversial in preventing some infections.

## Conclusions

Our study showed that for *S. aureus* strains isolated from skin, soft tissue, and bone related infections, resistance to antibiotics depends on the origin of the strain. We attempted to perform a correlation analysis between toxin production, resistance to antibiotics, and the origin of samples. The *S. aureus* strains examined in this study produced a variety of toxins, with PVL, one of the most severe *S. aureus* toxins, being the most common amongst all of the strains. Overall, it is desirable to integrate to the current morphological and biochemical diagnostic analysis with virulence factor screening to accurately diagnose infectious disease mediated by *S. aureus*. This integrated diagnostic strategy will help to efficiently treat patients affected by pathogenic *S. aureus* strains. This study concerning skin, soft tissue, and bone related infections should be extended to include other types of infections in Benin.

## Methods

### Ethics statement

Ethical clearance was obtained from the Ministry of Public health of Benin Republic under protocol number: **2959/MSP/DC/SG/DRH/SPREA-05-2002**. But it was important to notice that, the strains were de-identified and analyzed anonymously and the strains, not a human, were studied.

### Samples collection

Clinical *Staphylococcus aureus* samples were collected from patients with skin, soft tissue at the National University Hospital of Cotonou (Benin) for various bacteriological screenings in routine, from November 2009 to March 2011. The incidence of secondary infections in Burili ulcer is unknown; antibiotics may be frequently prescribed for this indication. It is equally unknown which bacteria these antibiotics should target and what the sensitivity of these bacteria is. So the samples from Burili ulcer were screened for *S. aureus*. Theses samples were carried out during a prospective study made in a village of Lalo in Benin. Osteomyelitis and pyomyositis samples were collected during a prospective study made in a Hlagba Ouassa village in Benin. So these strains are considered as community strains and the others sample were isolated in hospital as stated previously.

### *S. aureus*’ identification

Standard microbiological methods for identification of microorganisms were applied. All swabs were inoculated onto mannitol salt agar, incubated at 37°C and inspected visually for three days. Any suspected colony was subcultured on tryptic soy agar (bioMérieux) and identified by subsequent Gram staining, catalase test and Slidex Staph Plus (bioMérieux) and the coagulase test with the rabbit plasma [64]. Bacterial identification was performed by colony isolation on sheep blood agar plates and the automated Vitek 2 system.

### Antibiotic susceptibility

Antimicrobial susceptibility was determined by the disc diffusion method of Kirby-Bauer on agar Mueller-Hinton (bioMérieux, Marcy l'Etoile, France) as recommended by the Antibiogram Committee of the French Microbiology Society (CASFM) [65]. After 24 h at 37°C, the zone of inhibition was measured. Susceptibility to vancomycin was confirmed by broth microdilution methodology as described by the Clinical and Laboratory Standards Institute (CLSI) [66]. For susceptibility to oxacillin, an inoculum of 10^7^ CFU/ml was prepared and the plate was incubated at 37°C for 24 hours on Mueller-Hinton agar + 2% NaCl. Antibiotic disks were obtained from Biorad, Marne la Coquette, France.

The 17 tested antibiotics were: benzyl penicillin (10 UI), oxacillin (5 μg), cefoxitin screen (30 μg), gentamicin (10 UI), tobramycin (10 μg), kanamycin (30 μg), vancomycin (30 μg), teicoplanin (30 μg), fusidic acid (10 μg), fosfomycin (50 μg), rifampicin (30 μg), trimethoprim/sulfamethoxazole (1.25/23.75 μg), erythromycin (15 μg), lincomycin (30 μg), pristinamycin (15 μg), linezolid (30 μg) and tetracyclin (30 UI).

### Toxin detection

#### Phenotypic detection of toxins

For the phenotypic detection of toxins radial gel immunodiffusion was performed. The production of Panton-Valentine Leukocidin (PVL) and epidermolysins A (ETA) and B (ETB) were evidenced from culture supernatants after 18 h of growth in Yeast Casamino-acid Pyruvate (YCP) medium [67] by radial gel immunodiffusion in 0.6% (wt/vol) agarose with component-specific rabbit polyclonal and affinity-purified antibodies [68,69].

#### Genotype detection of toxins

Presence of genes encoding for the 12 toxins, for which we don’t have antibody, was detected by Multiplex PCR using specific primers (Table 1) previously used for [70]. Then, the genes encoding for enterotoxins A (*sea*), B (*seb*), C (*sec*), D (*sed*), E (*see*), G (*seg*), H (*seh*), I (*sei*) and *tsst* were analyzed. Additionally, genes encoding PVL, ETA and ETB were also detected. Briefly, total DNA was purified using QIAamp® DNA Mini Kit (Qiagen, GmbH, Germany) with a Gene Amp® PCR System 9700 (Perkin-Elmer, Norwalk, USA) and amplified in a total volume of 50 μl containing 25 pmoles of each primer, 50 ng of total DNA, 1.5 mM MgCl_2_, 200 μM of dNTP mixture, 1× PCR reaction Buffer and 5 units of Taq™ DNA polymerase (Invitogen™). The thermal cycling conditions included an initial denaturation step (2 min at 92°C) followed by 35 cycles of amplification comprising three steps: 2 min denaturation for 92°C, 1 min annealing at 50°C, 2 min extension at 72°C. The reaction was terminated with 3 min extension at 72°C. PCR products were analysed by electrophoresis through 1.4% (wt/vol) agarose gel (Euromedex, Mundolsheim, France).

**Table 1:** Primers sequences used in this study for the detection of genes encoding toxins

**Primers**	**Sequences**	**Polarity**	**Expected fragment (base pair)**	**Accession N°**
***eta***	5′-GCAGGTGTTGATTTAGCATT-3′	Sens	93	A8658-043
	5′-AGATGTCCCTATTTTTGCTG-3′	Antisens		A8658-044
***etb***	5′-ACCCCTGTTCCCTTATCATC-3′	Sens	226	A8659-036
	5′-GTTTTTGGCTGCTTCTCTTG-3′	Antisens		A8659-037
***lpv***	5′-AAAATGCCACTGTTATCCAGAGGTA-3′	Sens	433	A8658-029
	5′-TTTGCAGCGTTTTGTTTTCG-3′	Antisens		A8658-030
***sea***	5′-ATGGTTATCAATGTGCGGGTGIIIIICCAAACAAAAC-3′	Sens	520	A8658-031
	5′-TGAATACTGTCCTTGAGCACCAIIIIIATCGTAATTAAC-3′	Antisens		A8658-032
***seb***	5′-TGGTATGACATGATGCCTGCACIIIIIGATAAATTTGAC-3′	Sens	163	A8658-033
	5′-AGGTACTCTATAAGTGCCTGCCTIIIIIACTAACTCTT-3′	Antisens		A8658-034
***sec***	5′-GATGAAGTAGTTGATGTGTATGGATCIIIIIACTATGTAAAC-3′	Sens	283	A8658-035
	5′-AGATTGGTCAAACTTATCGCCTGGIIIIIGCATCATATC-3′	Antisens		A8658-036
***sed***	5′-CTGAATTAAGTAGTACCGCGCTIIIIIATATGAAAC-3′	Sens	384	A8658-037
	5′-TCCTTTTGCAAATAGCGCCTTGIIIIIGCATCTAATTC-3′	Antisens		A8658-038
***see***	5′-CTTACCGCCAAAGCTGTCG-3′	Sens	159	A8658-039
	5′-GTCCACTTGTAAATGGTAGCGAGAA-3′	Antisens		A8658-040
***seg***	5′-AATTATGTGAATGCTCAACCCGAT-3′	Sens	408	HA01804615
	5′-CTTTAGTGAGCCAGTGTCTTGCTTTG-3′	Antisens		HA01804616
***seh***	5′- CATCTACCCAAACATTAGCACC-3′	Sens	222	HA01804617
	5′-AGAAATCAAGGTGATAGTGGCAA-3′	Antisens		HA01804618
***sei***	5′-AGGCGTCACAGATAAAAACCTACCIIIIICAAATCAACTC-3′	Sens	454	HA01804619
	5′-ACAAGGACCATTATAATCAATGCCIIIIITATCCAGTTTC-3′	Antisens		HA01804620
***tsst-1***	5′-ACCCCTGCCTTTCCATCATC-3′	Sens	209	A8658-041
	5′-TTTTCAGTATTTGTAACGCC-3′	Antisens		A8658-042

### Staphylococcal enterotoxins production (SEA, SEB, SEC, SED, SEE, SEG, SEH, SEI) by Bio-Plex Assay (xMAP Multiplex assay)

The centrifuged supernatant (3ml) of *S. aureus* grown on BHI at 37°C (night) was recovered and diluted ½ in TBS-Tween20-IgG rabbit nonspecific at 100μg/mL, and incubated for 30 min at room temperature. The Bio-Plex assays consisted of three incubation steps that were performed into flat-bottom Multiscreen microplates (pores diameter = 1.2 μm, Millipore) according to the previous describe method [71]. Any steps were separated by three washes into TBS-Tween 20.

### Statistical analysis

Microsoft Excel Spreadsheet has been used for data processing. For comparison tests of positive isolates of each patient group, we used the Student T test, and the Fischer’s test for lower number series (GraphPad Prism 5). P < 0.05 was considered statistically significant.

## Competing interests

Authors declare no conflict of interest.

## Authors’ contributions

Conception and design of the study: LB-M and GP. Acquisition of data: HS, AT-A, WM, YB, HB. Analysis and interpretation of data: LB, GP, YS. Drafting the article: LB-M, SOK, and HS. Revising it critically for important intellectual content: LB-M, GP, SOK, YS. Final approval of the version to be submitted: All the co-authors. All authors read and approved the final manuscript.
